# Head-to-Head Comparison of the Incremental Predictive Value of The Three Established Risk Markers, Hs-troponin I, C-Reactive Protein, and NT-proBNP, in Coronary Artery Disease

**DOI:** 10.3390/biom10030394

**Published:** 2020-03-04

**Authors:** Julius Nikorowitsch, Francisco Ojeda, Karl J. Lackner, Renate B. Schnabel, Stefan Blankenberg, Tanja Zeller, Mahir Karakas

**Affiliations:** 1 Clinic of Cardiology, University Heart and Vascular Center, 20251 Hamburg, Germany; J.Nikorowitsch@uke.de (J.N.); f.ojeda-echevarria@uke.de (F.O.); r.schnabel@uke.de (R.B.S.); s.blankenberg@uke.de (S.B.); t.zeller@uke.de (T.Z.); 2 Department of Laboratory Medicine, University Medical Center, Johannes Gutenberg University, 55122 Mainz, Germany; Karl.Lackner@unimedizin-mainz.de; 3 German Center for Cardiovascular Research (DZHK), Partner Site Rhein-Main, 55131 Mainz, Germany; 4 German Center for Cardiovascular Research (DZHK), Partner Site Hamburg, 23538 Lübeck, Kiel, Hamburg, Germany

**Keywords:** N-terminal pro-brain natriuretic peptide, high-sensitivity troponin I, high-sensitivity C-reactive protein, coronary artery disease, prognosis

## Abstract

Risk stratification among patients with coronary artery disease (CAD) is of considerable interest to potentially guide secondary preventive therapies. Cardiac troponins as well as C-reactive protein (hsCRP) and natriuretic peptides have emerged as biomarkers for risk stratification. The question remains if one of these biomarkers is superior in predicting adverse outcomes. Thus, we perform a head-to-head comparison between high-sensitivity troponin I (hsTnI), hsCRP, and N-terminal pro-brain natriuretic peptide (NT-proBNP) in patients with CAD. Plasma levels were measured in a cohort of 2193 patients with documented CAD. The main outcome measures were cardiovascular (CV) death and/or nonfatal myocardial infarction (MI). During a median follow-up of 3.8 years, all three biomarkers were associated with cardiovascular death and/or MI. After adjustments for conventional cardiovascular risk factors, the hazard ratio (HR) per standard deviation (SD) for the prediction of CV death and/or nonfatal MI was 1.39 [95% CI: 1.24–1.57, *p* < 0.001] for hsTnI, 1.41 [95% CI: 1.24–1.60, *p* < 0.001] for hsCRP, and 1.64 [95% CI: 1.39–1.92, *p* < 0.001] for NT-proBNP. However, upon further adjustments for the other two biomarkers, only NT-proBNP was still associated with the combined endpoint with an HR of 1.47 [95% CI: 1.19–1.82, *p* < 0.001]. Conclusively, NT-proBNP is reliably linked to CV death and MI in patients with CAD and provides incremental value beyond hsCRP and hsTnI.

## 1. Introduction

Stratification for subsequent coronary events among patients with coronary artery disease (CAD) is of considerable interest due to the potential to guide secondary preventive therapies [1,2]. Several biomarkers have emerged as prediction tools for future adverse events in patients with coronary heart disease.

N-terminal pro-brain natriuretic peptide (NT-proBNP) is the preferred biomarker for the detection of heart failure [3]. However, elevated levels of NT-proBNP are also associated with long-term adverse cardiac events in the general population and in patients with CAD [4,5]. In fact, in the general population, not only natriuretic peptides but high-sensitivity C-reactive protein (hsCRP) and high-sensitivity troponins (hsTnI) were also among the biomarkers with the highest predictive value for adverse cardiovascular events [6]. Furthermore, all three biomarkers proofed prognostic for cardiovascular events, especially in patients with coronary artery disease [7,8,9]. However, it is unknown if one of these biomarkers is superior to the others in risk prediction. If so, this biomarker could potentially qualify for routine risk assessment in the high-risk population of patients with coronary heart disease.

Therefore, it is the aim of this study to perform a head-to-head comparison of hsTnI, hsCRP, and NT-proBNP as prognostic biomarkers for the prediction of cardiovascular death and myocardial infarction in a cohort of 2193 patients with angiographically documented CAD.

## 2. Materials and Methods

### 2.1. Study Population

In the AtheroGene cohort, a total of 3800 patients who underwent coronary angiography at the Department of Medicine II of the Johannes Gutenberg-University Mainz or the Bundeswehr-Zentralkrankenhaus Koblenz between June 1999 and March 2000 were recruited [10,11]. Patients’ exclusion criteria were as follows: evidence of hemodynamically significant valvular heart disease, surgery, or trauma within the previous month; known cardiomyopathy; known cancer; febrile conditions; or use of oral anticoagulant therapy within the previous four weeks. Patients with missing information on the clinical presentation, missing laboratory measurements, or lack of information on the cause of death were excluded, resulting in a subgroup of 3423 participants. Additionally, missing samples and low sample volumes led to exclusion. Thus, the analyses were performed in 2193 subjects. Baseline characteristics were not relevantly different between the stable angina pectoris (SAP) and the acute coronary syndrome (ACS) cohort.

The study was conducted according to the Declaration of Helsinki and approved by the Ethic Board of the Johannes Gutenberg-University Mainz and the Physicians’ chamber of the State Rhineland-Palatinate (Germany) under the number 837.057.99. All participants gave written informed consent.

### 2.2. Data Collection

At baseline, all participants were subject to a standardized questionnaire containing sociodemographic information and medical history. In addition, information was taken from the patients’ hospital charts. CAD was diagnosed if the coronary angiogram showed at least one stenosis >30% in a major coronary artery. Unstable angina was diagnosed according to Braunwald [12]. This included new onset of severe or accelerated angina, or angina at rest. Acute myocardial infarction (AMI) was either ST-segment elevation with significant elevation of at least 2 mV in at least two contiguous leads, or nonST-elevation myocardial infarction based on clinic and positive in-house troponin concentrations. The final diagnosis of ACS (UA, NSTEMI, or STEMI) or SAP, which included patients presenting with chest pain not fulfilling the ACS criteria, was made retrospectively based on the judgment of two physicians, with access to the history and nature of the presenting symptoms, medical history, results of physical examination, and all of the medical records available from index hospitalization (including the results of troponin testing).

Arterial hypertension was defined as mean blood pressure of 140 mmHg (systolic) over 90 mmHg (diastolic). Subjects taking antihypertensive medication were also classified as having arterial hypertension, even if the blood pressure was controlled. Smokers were defined as patients who had smoked in the past 40 years, and nonsmokers as those who ceased smoking more than 40 years ago or who had never smoked. Diabetes mellitus was defined as patients taking oral blood glucose lowering therapy or substituting insulin. Dyslipidemia was defined according to guidelines of the time of the start of the study as an LDL/HDL ratio of >3.5. Body Mass Index (BMI) was calculated as weight in kilograms divided by the height in meters squared (kg/m^2^).

Median follow-up after discharge was 3.8 years. Information was obtained from the patients using a mailed standardized questionnaire. Information regarding adverse cardiovascular disease (CVD) events and treatment since discharge from the in-hospital rehabilitation clinic was obtained from the primary care physicians also by means of a standardized questionnaire. If a subject had died during follow-up, the death certificate was obtained from the local Public Health Department and the main cause of death was coded according to the International Classification of Diseases (ICD-9 pos. 390–459: ICD-10 pos. I0–I99 and R57.0). Adverse CVD events were defined either as CVD as the main cause of death (as stated in the death certificate), or nonfatal myocardial infarction (MI). All nonfatal adverse events were reported by the primary care physicians.

### 2.3. Laboratory Methods

Blood samples were obtained before angiography and application of heparin in a fasting state. EDTA and serum samples were stored after centrifugation and aliquotation at −80 °C for future laboratory analysis in all patients.

Plasma levels of hsTnI were determined using the Architect immunoassay (Abbott Diagnostics, ARCHITECT i1000SR). The limit of detection for the assay was 1.9 (range, 0–50,000) ng/L. The inter coefficient of variation (CV) was 2.88%, and the intra CV was 6.69%.

C-reactive protein was determined by a highly sensitive, latex particle-enhanced immunoassay (detection range of 0 to 20 mg/L, Roche Diagnostics, Mannheim, Germany). The measurement of NT-proBNP was performed on the ELECSYS 2010 by electrochemiluminescence sandwich immunoassay (ECLIA, Roche Diagnostics, Mannheim, Germany). The analytical reporting range is indicated as 5–35,000 ng/L. The lowest concentration detectable with an inter-assay CV of 20%, the functional assay sensitivity, is <50 ng/L. The interassay imprecision is 2.2–5.8% for the luminescence immunoassay. All biomarkers were measured in a blinded fashion.

### 2.4. Statistical Methods

The study population was described with respect to various sociodemographic and medical characteristics using quartiles for continuous variables and absolute and relative frequencies for binary variables. Differences between the ACS and SAP groups were tested using the Mann–Whitney test for continuous variables and the chi-squared test for binary variables. Spearman correlation coefficients were calculated to describe unadjusted associations of the three biomarkers with conventional cardiovascular risk factors and with each other. To examine adjusted associations of the three markers with conventional cardiovascular risk factors, each marker was linearly regressed after log-transformation on the following variables: age, sex, BMI, diabetes, smoking status, dyslipidemia, and hypertension. To display survival curves produced by the Kaplan–Meier method, subjects were grouped according to tertiles of biomarkers. The *p*-value displayed on the graphics is for the log-rank test (testing that the null hypothesis of equality of survival curves versus at least two of the curves is different). The association of circulating biomarker levels, used after log-transformation, with cardiovascular mortality and nonfatal MI during follow-up was assessed by Cox proportional hazards analyses adjusted for age (years) and sex (model 1). In additional models, the age- and sex-adjustment was extended to conventional cardiovascular risk factors (BMI, diabetes, smoking status, dyslipidemia, hypertension (model 2)), and the respective two other biomarkers (model 3).

The assumptions for using Cox regression were tested, and no evidence of violation was found. Additionally, the C-index and the categorical net reclassification index (NRI) were calculated using the category five-year event probabilities and were used to compare the performance of different models. The following risk categories were used for the NRI computations: <1%, 1–5%, 5–10%, and >10%. Multiple imputation was used to fill the missing values. It was performed by using Multivariate Imputations by Chained Equations (MICE), as proposed by Buuren and Groothuis-Oudshoorn [13]. All computations were performed with R version 3.6.1 (http://www.r-project.org/). A *p*-value of <0.05 was considered statistically significant.

## 3. Results

A total of 2193 individuals with evident CAD and available hsTnI measurements were included in this analysis. Table 1 presents the main sociodemographic and laboratory characteristics of the individuals at baseline, stratified according to the diagnosis at presentation ACS or SAP. Baseline characteristics were not relevantly different between the SAP and the ACS cohort. The mean age of SAP patients was 64 years, and similarly 63 years in ACS. The majority of participants were male—77.1% in the SAP cohort and 76.2% in the ACS cohort. As expected, in patients with ACS, levels of hsTnI, hsCRP, and NT-proBNP were higher and hypertension was more frequent than in SAP patients. Dyslipidemia was present in 77.4% of the SAP cohort, compared to 64.4% in the ACS cohort.

During a median follow-up of 3.8 years, 231 (10.5%) cardiovascular deaths or nonfatal MI were documented, 114 (8.4%) among SAP patients and 117 (14%) in the ACS cohort.

In order to assess the correlation of each hsTnI, hsCRP, and NT-proBNP with common cardiovascular risk factors such as diabetes, smoking status, and arterial hypertension, as well as amongst each other, Spearman correlation coefficients (R) were calculated (Table 2). The strongest associations were observed for the biomarkers with each other, in particular for NT-proBNP and hs-TnI with a correlation coefficient of 0.6 (*p* < 0.001). Weaker but still significant correlations were observed for diabetes and age with all three biomarkers.

Linear regression demonstrated significant associations of the three biomarkers with most of the mentioned common cardiovascular risk factors. However, an R^2^ of 0.1 for NT-proBNP was higher than that of hsCRP and hsTnI, each of which had an R^2^ of 0.06, suggesting a larger influence of the cardiovascular risk factors on NT-proBNP levels than on hsCRP levels and hsTnI levels.

**Figure 1:**
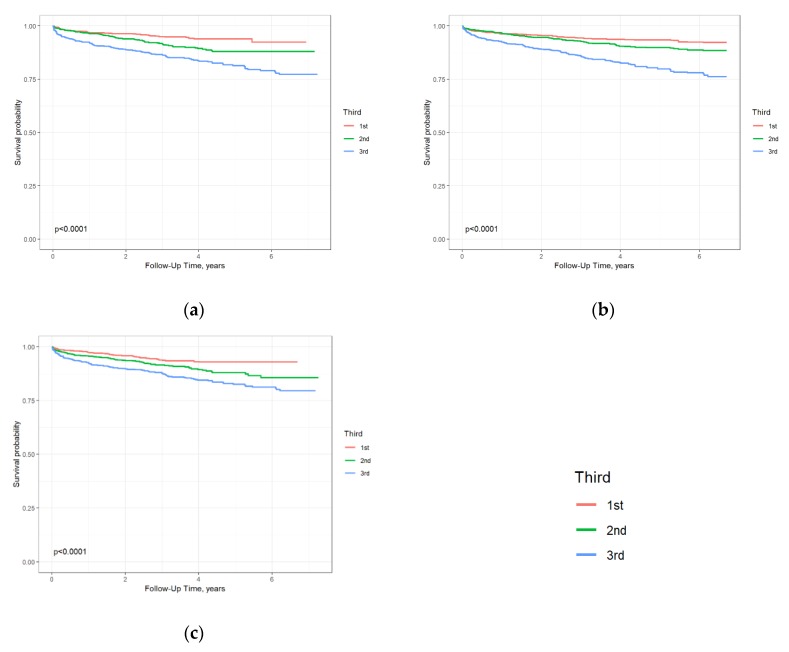
Survival curves for cardiovascular (CV) death and/or acute myocardial infarction (MI) according to tertiles of levels of hsTnI: (**a**) (1st tertile up to 5.20 ng/L; 2nd tertile up to 24.63 ng/L), NT-proBNP, (**b**) (1st tertile up to 132.74 pg/mL; 2nd tertile up to 418.14 pg/mL) and hsCRP, and (**c**) (1st tertile up to 2.04 mg/L; 2nd tertile up to 6.35 mg/L) in all. The *p*-values are for the logrank test.

Survival curves according to tertiles of circulating hsTnI levels (*p* < 0.001), hsCRP levels (*p* < 0.0001), and NT-proBNP levels (*p* < 0.0001) evidenced the prognostic relevance of the three biomarkers for cardiovascular death and/or nonfatal MI (Figure 1).

In Cox regression analyses with adjustment for sex and age (model 1), the hazard ratio (HR) per standard deviation (SD) for the prediction of cardiovascular death and/or MI during follow-up was 1.37 [95% CI: 1.23–1.53, *p* < 0.001] for hsTnI, 1.41 [95% CI: 1.24–1.60, *p* < 0.001] for hsCRP, and 1.65 for NT-proBNP [95% CI: 1.40–1.93, *p* < 0.001] (Table 3).

After further multivariate adjustment for conventional cardiovascular risk factors (model 2), all three biomarkers still enabled a strong prediction of future cardiovascular death and/or MI, with an HR per SD of 1.39 [95% CI: 1.24–1.57; *p* < 0.001] for hsTnI, 1.41 [95% CI: 1.24–1.60, *p* < 0.001] for hsCRP, and 1.64 [95% CI: 1.39–1.92; *p* < 0.001] for NT-proBNP. However, further adjustments for the other two biomarkers, respectively, led to an attenuation of the predictive value of both hsTnI with an HR per SD of 1.14 [95% CI: 0.95–1.38, *p* = 0.16] as well as hsCRP with an HR of 1.16 per SD [95% CI: 0.99–1.35, *p* = 0.064]. Only for NT-proBNP the predictive value remained significant for cardiovascular death and/or MI beyond the other two biomarkers, with an HR per SD of 1.47 [95% CI: 1.19–1.82, *p* < 0.001]. In addition, NT-proBNP allowed for a strong prognostication of the outcome cardiovascular death, after full adjustments (model 3), with an HR per SD of 2.42 [95% CI: 1.86, 3.15, *p* < 0.001] (Table 4).

Accordingly, an NRI of 0.282 [95% CI: 0.114–0.450] as well as a C-index difference of 0.053 [95% CI: 0.017–0.089, *p* = 0.0038] proofed an additional prognostic power of NT-proBNP beyond both cardiovascular risk factors and the biomarkers hsTnI and hsCRP. In contrast, both hsTnI with an NRI of 0.011 [95% CI: −0.064–0.086] and a C-index difference of 0.002 (*p* = 0.56), and hsCRP with an NRI of 0.024 [95% CI: −0.042–0.091] and a C-index difference of 0.006 n (*p* = 0.26), showed no significant improvement of discrimination.

## 4. Discussion

### 4.1. NT-proBNP Levels Progosticate CV Death and MI Beyond hsCRP and hsTnI

The present study aimed for the head-to-head comparison of three established blood-based cardiovascular biomarkers. Therefore, we assessed the prognostic value of circulating levels of hsTnI, NT-proBNP, and hsCRP for cardiovascular death and secondary myocardial infarction, as well as cardiovascular death alone in a cohort of patients with documented CAD. Our data revealed a statistically significant impaired prognosis with increasing levels of all three biomarkers independent of traditional cardiac risk factors. However, in a head-to-head comparison, only NT-proBNP yielded additional prognostic value beyond the other two biomarkers.

The prognostic relevance of troponins, hsCRP, and natriuretic peptides, in patients with CAD, has been established before [8,9,10,11]. However, to the best of our knowledge, this is the first study to evaluate and demonstrate a significant additional prognostic value of NT-proBNP beyond hsCRP and hsTnI in patients with coronary artery disease.

In the general population, troponin, hsCRP, and NT-proBNP were the three biomarkers best predicting cardiovascular adverse events among 30 tested biomarkers during a follow-up of ten years [6]. The combined use of NT-proBNP and hsCRP improves long-term risk prediction of mortality in patients with stable coronary heart disease [14]. NT-proBNP, in contrast to contemporary-sensitive Troponin T, improved risk prediction and classification compared to the Framingham Risk Score and the pooled Cohort Risk Equation in 5592 asymptomatic participants of the Multi-Ethnic Study of Atherosclerosis [15]. Similarly, compared to a conventional troponin assay, NT-proBNP was superior in risk prediction in a cohort of 987 patients with stable coronary heart disease [9].

Elevated NT-proBNP levels not only precede the development of arterial hypertension, a major risk factor for CAD, but were inversely associated with myocardial perfusion reserve assessed by cardiac magnetic resonance before and after the administration of adenosine in 184 asymptomatic individuals, suggesting an association of NT-proBNP levels and microvascular dysfunction [16,17].

Accordingly, our study demonstrates that NT-proBNP levels not only outperform the prognostic value of hsCRP but also outperform hsTnI in risk estimation.

Consequently, the biomarker NT-proBNP seems to be the most appropriate biomarker for mid-term risk estimation in CAD patients compared to the biomarkers hsCRP and hsTnI.

### 4.2. Pathophysiologic Considerations

NT-proBNP is a cleavage product of proBNP that is released by the myocardium in response to left ventricular wall stress [18,19]. In patients with stable coronary artery disease, relevant coronary stenosis may cause transient myocardial ischemia, thereby probably increasing wall stress and releasing natriuretic peptides [20]. Accordingly, recent studies found a correlation of natriuretic peptides with ischemic burden, hence the amount of affected myocardium [21]. The amount of ischemic myocardium is known to be predictive for adverse outcomes in CAD patients [22]. Acute coronary syndromes imply malfunctioning myocardium, hence increasing regional wall stress. The levels of natriuretic peptides therefore represent the functional consequences of myocardial damage. Conversely, levels of hsCRP, reflecting systemic inflammation, do not seem to be equally predictive for adverse events in CAD patients. Similarly, hsTnI, which is released from acutely injured myocardium, appears to be inferior to NT-proBNP in risk prediction [23]. After all, hemodynamic consequences as a result of ventricular dysfunction caused by CAD, which are best represented by NT-proBNP, might be the best parameter for the prediction of future adverse events. Therefore, it seems plausible that NT-proBNP plays an important role in risk stratification in CAD patients and might be superior to hsCRP and hsTnI.

### 4.3. Future Perspectives

Risk stratification for secondary adverse cardiovascular events in patients with coronary heart disease will be increasingly important in the future, especially for individually tailored therapy. Here, we suggest NT-proBNP as a candidate biomarker for risk prediction in patients with CAD, as part of a risk prediction panel including additional biomarkers. Microvesicles, which are small extracellular membrane particles shed by activated and apoptotic cells, have emerged as promising biomarkers for risk prediction not only in many inflammation-associated disorders in general but especially in cardiovascular diseases [24,25]. Although specific roles of each phenotype still remain to be further evaluated, a multipanel approach including NT-proBNP might facilitate risk prediction in CAD patients [26]. Such multimarker approaches should be further evaluated, and maybe in addition to other risk factors, reliable risk prediction tools could be developed.

### 4.4. Study Limitations

Our study has some limitations that need to be addressed. As typical for CHD populations, women were clearly underrepresented. Although we had a large sample of ACS patients, fatal CVD events were limited in this study population. Furthermore, our study population only represents patients with CAD who underwent coronary angiography. This population might differ from CAD patients without the need for coronary intervention. Therefore, the predictive value of NT-proBNP, hsTnI, and hsCRP levels should also be assessed in those patients. As blood samples have been stored for up to 20 years at −80 °C, long-term storage might affect measurements of hsTnI, NT-proBNP, or hsTnI. Additionally, participant acquisition was conducted from 1999 to 2000, so that altered therapy regimen might influence the transferability of the results to contemporary patient populations. Finally, net reclassification indices, as well as Cox regression analyses, should be interpreted with caution, due to possible misinterpretations and over-adjustment.

## 5. Conclusions

Our study demonstrates that hsTnI, hsCRP, and NT-proBNP levels prognosticate cardiovascular mortality and nonfatal MI in patients with coronary artery disease. However, only NT-proBNP yields an additional prognostic value beyond the two other markers.

## Figures and Tables

**Table 1:** Characteristics of the study patients with coronary heart disease.

	SAP	ACS	*p*-Value
n	1,356	837	
Age (years) *	64.0 (56.0, 70.0)	63.0 (55.0, 70.0)	0.61
Male No. (%)	1045 (77.1)	638 (76.2)	0.69
BMI (kg/m^2^) *	27.2 (25.0, 30.1)	27.1 (24.9, 29.7)	0.23
Diabetes No. (%)	318 (23.5)	171 (20.4)	0.11
Current smoker No. (%)	285 (21.0)	235 (28.1)	<0.001
Dyslipidemia No. (%)	1049 (77.4)	539 (64.4)	<0.001
Hypertension No. (%)	1108 (81.7)	574 (68.6)	<0.001
NT-proBNP (pg/mL) *#	178.4 (82.6, 458.7)	435.2 (165.5, 1426.0)	<0.001
hsCRP (mg/L) *#	2.7 (1.3, 6.0)	6.2 (2.4, 17.9)	<0.001
hsTnI (ng/L)	5.8 (3.3, 12.6)	142.8 (10.4, 2754.2)	<0.001

SAP = stable angina pectoris, ACS = acute coronary syndrome, BMI = body mass index, NT-proBNP = N-terminal prohormone of brain natriuretic peptide, hsCRP = high-sensitivity C-reactive protein, and hsTnI = high-sensitivity troponin I. * Median 25th and 75th quartile cut-point. # only available in a subset of patients.

**Table 2:** Spearman correlations of selected variables with hsTnI, hsCRP, and NT-proBNP.

Variable	hsTnI	hsCRP	NT-proBNP
Age	0.10 (*p* < 0.001)	0.08 (*p* < 0.001)	0.29 (*p* < 0.001)
Male	0.04 (*p* = 0.072)	−0.11 (*p* < 0.001)	−0.13 (*p* < 0.001)
BMI	0.00 (*p* = 0.850)	0.10 (*p* < 0.001)	−0.05 (*p* = 0230)
Diabetes	0.05 (*p* = 0.020)	0.05 (*p* = 0.021)	0.08 (*p* < 0.001)
Smoker	0.13 (*p* < 0.001)	0.13 (*p* < 0.001)	0.01 (*p* = 0.810)
Dyslipidaemia	−0.10 (*p* < 0.001)	−0.08 (*p* < 0.001)	−0.06 (*p* = 0.084)
Hypertension	−0.10 (*p* < 0.001)	−0.04 (*p* = 0.055)	0.00 (*p* = 0.940)
NT-proBNP	0.60 (*p* < 0.001)	0.39 (*p* < 0.001)	1
CRP	0.44 (*p* < 0.001)	1	0.39 (*p* < 0.001)
hsTnI	1	0.44 (*p* < 0.001)	0.6 (*p* < 0.001)

**Table 3:** Association of circulating biomarkers with cardiovascular death and/or MI during follow-up. The hazard ratios (HRs) are per standard deviation.

	Model	HR (95% CI)	*p*-Value	N	N Events
hsTnI	1	1.37 (1.23, 1.53)	<0.001	2193	231
	2	1.39 (1.24, 1.57)	<0.001	2193	231
	3	1.06 (0.90, 1.26)	0.470	2193	231
hsCRP	1	1.41 (1.24, 1.60)	<0.001	2193	231
	2	1.41 (1.24, 1.60)	<0.001	2193	231
	3	1.16 (0.99, 1.35)	0.064	2193	231
NT-proBNP	1	1.65 (1.40, 1.93)	<0.001	2193	231
	2	1.64 (1.39, 1.92)	<0.001	2193	231
	3	1.47 (1.19, 1.82)	<0.001	2193	231

HR = hazard ratio and CI = confidence interval. Model 1: adjusted for age, sex; Model 2: additionally adjusted for body-mass-index, diabetes, smoking status, dyslipidemia, and hypertension; Model 3: additionally adjusted for log(NT-proBNP) and log(hsCRP). Circulating biomarkers were used log-transformed.

**Table 4:** Association of circulating biomarkers with cardiovascular death during follow-up. The HRs are per standard deviation.

	Model	HR (95% CI)	*p*-Value	N	N Events
hsTnI	1	1.39 (1.19, 1.61)	<0.001	2193	123
	2	1.43 (1.22, 1.67)	<0.001	2193	123
	3	0.81 (0.65, 1.02)	0.077	2193	123
hsCRP	1	1.60 (1.35, 1.89)	<0.001	2193	123
	2	1.63 (1.38, 1.94)	<0.001	2193	123
	3	1.26 (1.02, 1.55)	0.031	2193	123
NT-proBNP	1	2.37 (1.92, 2.91)	<0.001	2193	123
	2	2.39 (1.93, 2.95)	<0.001	2193	123
	3	2.42 (1.86, 3.15)	<0.001	2193	123

Model 1: adjusted for age and sex; Model 2: additionally adjusted for body-mass-index, diabetes, smoking status, dyslipidemia, and hypertension; Model 3: additionally adjusted for log(NT-proBNP) and log(hsCRP). Circulating biomarkers were used log-transformed.
